# HAMMER: automated operation of mass frontier to construct *in silico* mass spectral fragmentation libraries

**DOI:** 10.1093/bioinformatics/btt711

**Published:** 2013-12-11

**Authors:** Jiarui Zhou, Ralf J. M. Weber, J. William Allwood, Robert Mistrik, Zexuan Zhu, Zhen Ji, Siping Chen, Warwick B. Dunn, Shan He, Mark R. Viant

**Affiliations:** ^1^College of Biomedical Engineering and Instrument Science, Zhejiang University, Hangzhou 310027, China, ^2^School of Biosciences, University of Birmingham, Birmingham, B15 2TT, UK, ^3^HighChem, Ltd., Leskova 11, 81104 Bratislava, Slovakia, ^4^Shenzhen City Key Laboratory of Embedded System Design, College of Computer Science and Software Engineering, ^5^School of Medicine, Shenzhen University, Shenzhen 518060, China and ^6^School of Computer Science, University of Birmingham, Birmingham, B15 2TT, UK

## Abstract

**Summary:** Experimental MS^n^ mass spectral libraries currently do not adequately cover chemical space. This limits the robust annotation of metabolites in metabolomics studies of complex biological samples. *In silico* fragmentation libraries would improve the identification of compounds from experimental multistage fragmentation data when experimental reference data are unavailable. Here, we present a freely available software package to automatically control Mass Frontier software to construct *in silico* mass spectral libraries and to perform spectral matching. Based on two case studies, we have demonstrated that high-throughput automation of Mass Frontier allows researchers to generate *in silico* mass spectral libraries in an automated and high-throughput fashion with little or no human intervention required.

**Availability and implementation:** Documentation, examples, results and source code are available at http://www.biosciences-labs.bham.ac.uk/viant/hammer/.

**Contact:** m.viant@bham.ac.uk

**Supplementary information:** Supplementary data are available at *Bioinformatics* online.

## 1 INTRODUCTION

Mass spectrometry (MS)-based metabolomics is a rapidly developing field that aims to detect and measure a variety of small biological molecules (metabolites) over a wide dynamic range (Dettmer *et al.*, 2007). Although thousands of metabolites are typically detected in an untargeted metabolomics study of a biological sample, their subsequent identification represents the most significant bottleneck in the discovery of new biochemical knowledge (Dunn *et al.*, 2013; Kind and Fiehn, 2010; Wishart, 2011). In many cases, multiple empirical formulae and/or putative chemical structures are reported for each observed mass feature (or more strictly mass-to-charge ratio, *m/z*). Multistage (MS^n^) mass spectrometry, which is an experimental technique to collect in-depth fragmentation data related to the chemical structure of metabolites, is often applied to increase accuracy and specificity in metabolite annotation and identification. However, experimental MS^n^ mass spectral libraries currently do not adequately cover the search space for all metabolites present in complex biological samples, as authentic chemical standards are not available for all metabolites. The generation of *in silico* MS^n^ libraries is anticipated to greatly improve the success rate of annotation of metabolites detected in metabolomics studies, when experimentally acquired MS^n^ data of authentic chemical standards are unavailable. Commercial software packages such as Mass Frontier and ACD/MS Fragmenter predict *in silico* fragmentation patterns to assist the interpretation of experimental MS^n^ data (Krauss *et al.*, 2010; Oppermann *et al.*, 2012). Additionally, a freely available tool named MetFrag has been developed previously (Wolf *et al.*, 2010). This tool selects candidate structures from a compound library based on the molecular ion *m/z* and generates *in silico* substructures to subsequently annotate fragment peaks. MetFrag does not provide the ability to create *in silico* patterns or libraries independent of the use of experimental data; however, it has been proven to be an efficient approach to annotate fragmentation mass spectra derived from MS experiments.

Mass Frontier is a well-known software package that has been used by more than a thousand academic institutions and companies throughout the world for the management, evaluation and interpretation of mass spectra. It includes three resources that assist the user to robustly predict *in silico* fragmentation patterns: a set of general fragmentation rules, a fragmentation library comprising ∼150 000 fragmentation mechanisms collected from the scientific literature and user-specified mechanisms. However, Mass Frontier cannot readily perform batch processing on the scale required to generate comprehensive *in silico* libraries for thousands of metabolites.

Here, we present a freely available software package, named HAMMER (High-throughput AutoMation of Mass frontiER), to automatically control Mass Frontier software to perform *in silico* fragmentation in an automated and high-throughput matter. This package allows the user to readily generate large-scale *in silico* fragmentation libraries and perform mass spectral matching to mass spectrometry-derived data.

## 2 METHODS AND IMPLEMENTATION

HAMMER is developed under the Python language and supports Windows 7 and XP (both 32 bit and 64 bit). Its software requirements include Java 6/7, Open Babel (O'Boyle *et al.*, 2011), Sikuli (Yeh *et al.*, 2009) and Mass Frontier 7. A desktop PC with 2.66 GHz CPU and 4 GB memory was used as the operating platform.

**Fig. 1.**
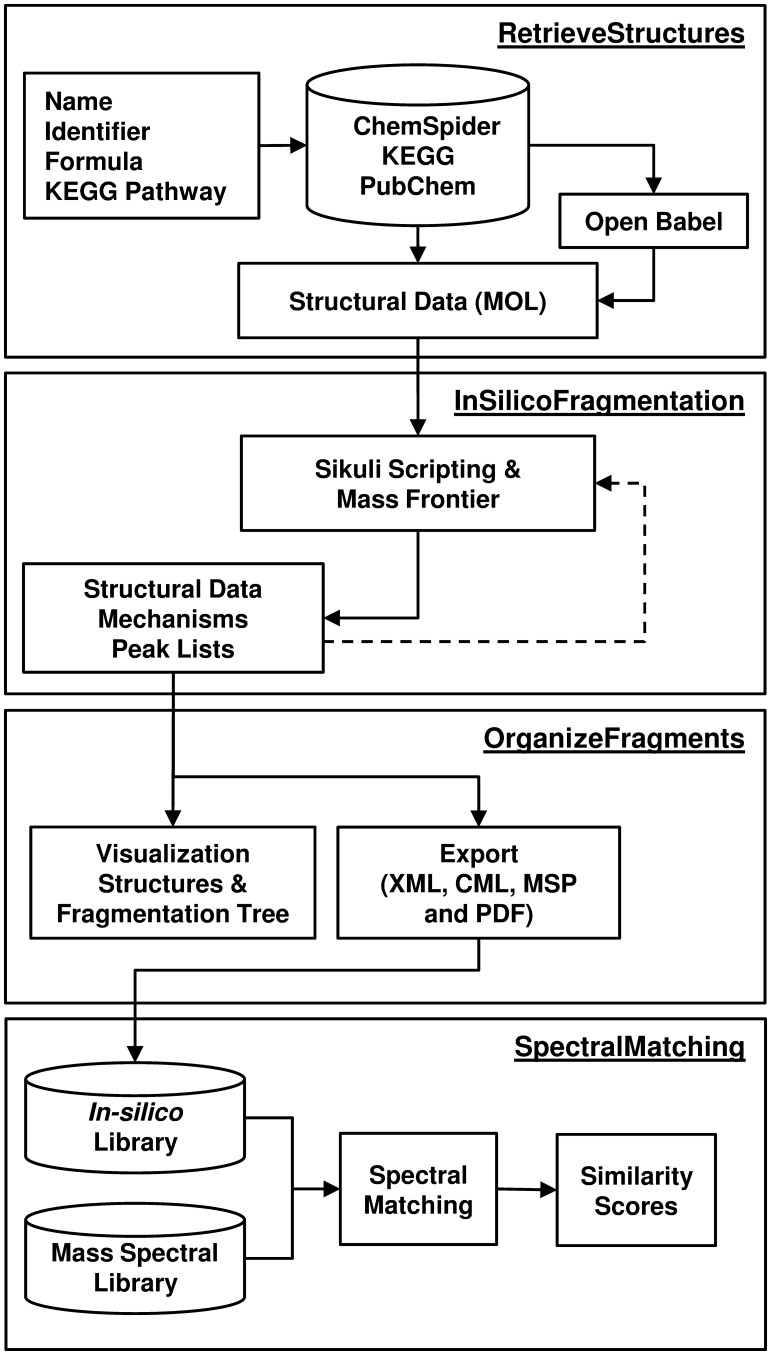
Workflow for HAMMER

As shown in Figure 1, HAMMER consists of four modules:

1. ***RetrieveStructures*** retrieves chemical structures from a compound database of interest such as ChemSpider, Kyoto Encyclopedia of Genes and Genomes (KEGG) and PubChem. The retrieval is based on a submitted plain text file that includes a list of compounds or chemicals (with specific names, database identifier or chemical formula). Additionally, metabolites within specific KEGG pathways can be downloaded based on a KEGG pathway identifier. Candidate entries found in the databases of interest are automatically downloaded, verified and converted into .mol format (required by Mass Frontier) or other formats such as SMILES, InChI or empirical formula. Open Babel is used to perform structure verification and conversion. A report is generated to summarize the information retrieved for each compound (e.g. number of candidates, database IDs and URL to the specific database entry).
2. ***InSilicoFragmentation*** automatically controls the operation of Mass Frontier by using the open-source visual scripting software Sikuli. Sikuli allows control of software when no application programming interface is available, which is the case for many commercial software packages. It readily uses image recognition of graphical user interface (GUI) elements to operate software. HAMMER contains a Sikuli standalone runtime. As a result, the *InSilicoFragmentation* module works without any setup, otherwise simple configuration is required. Two versions of *InSilicoFragmentation* are provided: Windows 7 and Windows XP. We have separated the GUI images (or patterns) and search regions from the source code itself. This allows the user to modify the script more easily if required (e.g. applying a different version of Mass Frontier, see online manual for details). The structure file (.mol) of each compound is separately imported into Mass Frontier, and *in silico* fragmentation is performed applying user-defined fragmentation settings (see online manual for details). Structural and *m/z* information for each fragment is collected for each *in silico* reaction and if required (i.e. for MS^n^) is used for the next fragmentation step. This use allows closed-loop operation of Mass Frontier to perform ‘multistage’ *in silico* fragmentation. *In silico* fragments, their corresponding unique *m/z* values, chemical structures (in each stage) and fragmentation mechanisms are exported for further analysis and visualization.
3. ***OrganiseFragments*** uses the *in silico* fragmentation results as its input. The fragments are organized systematically in separate folders according to the compound of interest, and a 2D chemical structure image file in.png format is generated for each fragment. The *in silico* fragmentation results are exportable in several formats, such as in extensible mark-up language and plain text formats, as well as chemical mark-up language and National Institute of Standards and Technology spectra library files (NIST-MSP) for further analysis. These formats are compatible with common mass spectrometry databases and software packages. The hierarchical relationships of the fragments are parsed to an extensible mark-up language file that can easily be accessed using scripting languages. Additionally, a hierarchical tree visualization (as a .pdf) is generated to allow visual comparison or interpretation.
4. ***SpectralMatching*** computes a score, using the pMatch algorithm, to evaluate the probability of a match between an experimental fragmentation spectrum and an *in silico* fragmentation pattern in a library (Ye et al., 2010). The pMatch algorithm is a spectral matching algorithm originally developed for mass spectrometry-based protein identification (see Supplementary Information for details of the algorithm). The SpectralMatching module uses MSP files (see previous module), experimental and *in silico*, as its input. For each experimental fragmentation spectrum, a report is generated, which includes detailed information on the matching and annotation.

## 3 CASE STUDIES

HAMMER was used to generate *in silico* fragmentation data for two distinct groups of compounds: (i) all 72 metabolites within the ‘phenylalanine metabolism’ KEGG pathway (i.e. map00360) and (ii) the top 200 most prescribed drugs in the USA in 2011 (RxList, 2011). These were selected to demonstrate the applicability and capability of HAMMER across diverse compound sets. Some drugs are composed of multiple compounds, and each compound was separately parsed and imported into Mass Frontier. Additionally, lower mass neutral and charged molecules/atoms (e.g. water and sodium) within these separated drug compounds were excluded from this demonstration dataset (see Supplementary Information for details). This resulted in 151 unique structures for the drugs dataset. See Supplementary Table S1 for specific settings used for both case studies. The run times for the phenylalanine and drug datasets were ∼8.5 and 14.5 h, respectively. On average, ∼3200 fragments were predicted for each compound (see Supplementary Tables S2–S4). Applying the defined Mass Frontier parameter settings, it is expected that MS*^n^* data are reported, where *n* > 2. This relatively high number of fragments illustrates the complexity and diversity of the fragmentation mechanisms. Although, these *in silico* fragments are predicted based on a set of general fragmentation rules and a fragmentation library comprising ∼150 000 fragmentation mechanisms collected from the scientific literature, the numbers of fragments produced are highly dependent on the complexity of the chemical structures and the parameter settings defined by the user (see Supplementary Information and online manual for details). *In silico* fragments may be reported, which are false positives; these fragments are not actually created in the MS fragmentation process, or are created but their low stabilities ensure that further and complete fragmentation or decomposition is observed before the ions are detected.

To assess the applicability of each *in silico* fragmentation library, five experimental fragmentation mass spectra were retrieved from MassBank to perform spectral matching [Supplementary Table S5, Case Study I: acetyl-CoA (KNA00207), capsaicin (WA001605), isobutyryl-CoA (PR100154), N-acetyl-L-phenylalanine (KO002200) and succinic acid (KZ000074). Case study II: amoxicillin (WA001751), digoxin (WA000563), meloxicam (WA002576), naproxen (WA000359) and prednisone (CO000368)] (Horai *et al.*, 2010). All of the 10 real fragmentation spectra were correctly identified based on the data present in these small *in silico* mass spectral libraries (Supplementary Table S6).

It is important to highlight that the parameter settings used in Mass Frontier to perform *in silico* fragmentation play an important role in the correct prediction of *in silico* fragments and mechanisms. Additionally, understanding the experimental parameters used to collect experimental data (e.g. difference in collision energy or type of fragmentation) is also important to maximize metabolite annotation. However, these two challenges are outside the scope of the work presented here.

Both case studies show that HAMMER allows researchers to readily generate *in silico* MS^n^ libraries and perform batch spectral matching of *in silico* mass spectral data to MS-derived data in an automated high-throughput fashion with minimal or no human intervention. In addition, we have shown with both case studies the value and applicability of visual scripting in the field of computational biology. We anticipate this software package will be used across a wide range of disciplines including metabolomics, organic synthetic chemistry and pharmaceutical research.

*Funding*: Royal Society International Exchanges 2011 NSFC funding (61211130120), National Natural Science Foundation of China (NSFC) Joint Fund with Guangdong (U1201256), NSFC funding (61171125 and 61001185), the UK Natural Environment Research Council (NE/I008314/1) and the Systems Science for Health initiative at The University of Birmingham.

*Conflict of Interest*: R.M. derives income from Mass Frontier sales.

## Supplementary Material

Supplementary Data
