# Supplementary material for: HAMMER: automated operation of mass frontier to construct in silico mass spectral fragmentation libraries
Source: Bioinformatics. 2013 Dec 11;30(4):581–3. doi: 10.1093/bioinformatics/btt711 (PMC3928522; doi:10.1093/bioinformatics/btt711)
Supplement: Supplementary Data [file supp_30_4_581__index.html]

HAMMER: Automated operation of Mass Frontier to construct in-silico mass spectral fragmentation libraries — HAMMER: automated operation of mass frontier to construct in silico mass spectral fragmentation libraries — HAMMER: automated operation of mass frontier to construct in silico mass spectral fragmentation libraries — Supplementary Data 

# HAMMER: automated operation of mass frontier to construct *in silico* mass spectral fragmentation libraries

## Supplementary Data

files

**Files in this Data Supplement:**

- Supplementary Data - pdf file
