# Supplementary material for: HAMMER: automated operation of mass frontier to construct in silico mass spectral fragmentation libraries
Source: Bioinformatics. 2013 Dec 11;30(4):581–3. doi: 10.1093/bioinformatics/btt711 (PMC3928522; doi:10.1093/bioinformatics/btt711)
Supplement: Supplementary Data [file supp_btt711_Bioinformatics_AppNote_Suppl_Info_rev_211113.pdf]

HAMMER: Automated operation of Mass Frontier to construct *in-silico* mass spectral fragmentation libraries

Jiarui Zhou[1,†], Ralf J. M. Weber[2,†], J. William Allwood[2], Robert Mistrik[4], Zexuan Zhu[5], Zhen Ji[5], Siping Chen[6], Warwick B. Dunn[2], Shan He[3] and Mark R. Viant[2,*]

[1]College of Biomedical Engineering and Instrument Science, Zhejiang University, Hangzhou 310027, China; [2]School of Biosciences and [3]School of Computer Science, University of Birmingham, Birmingham, B15 2TT, United Kingdom; [4]HighChem, Ltd., Leskova 11, 81104 Bratislava, Slovakia; [5]Shenzhen City Key Laboratory of Embedded System Design, College of Computer Science and Software Engineering, [6]School of Medicine, Shenzhen University, Shenzhen 518060, China;

[*]To whom correspondence should be addressed, [†]These authors made an equal contribution to this work.

Table S1: Mass Frontier parameters used to conduct automated *in-silico* fragmentation.

**Knowledge base**	General fragmentation rules, HighChem ESI Pos 2008* and HighChem Fragmentation Library
**Reaction steps**	7**
**Reactions limit**	20,000**
**Ionisation method**	$[M + H]^+$

* Includes the most prescribed drugs in Europe. This spectral library was manually annotated with the intention to improve the prediction for most common drugs.
** See online user manual section 3.2.2 for more details.

Table S2: Summary of *in-silico* fragmentation results for the two datasets presented more exhaustively in Tables S3 and S4.

Case Study	No. of Compounds	Unique Structures	Fragments Produced	Run Time (Sec)	Failed
**Phenylalanine metabolism KEGG pathway**	72	72	232618	29871	1*
**Top 200 prescribed drugs in USA in 2011*** **	200	151	489300	52459	3**

* No fragment(s) can be generated for compound KGID_C00084.

** No fragment(s) can be generated for compound CSID_54768, CSID_7843322, and CSID_5293370.

*** Some compounds contain more than one molecule, and will be split automatically. Lower mass neutral, charged molecules, and single elements were removed. If a molecule is exported from more than one compound, only one of them will be fragmented. The molecules will be verified using InChI, and, if no error can be found, exported in MOL format. Finally 151 qualified MOL files were sent to Mass Frontier for *in-silico* fragmentation. Validation steps described here are conducted automatically in HAMMER.

Table S3: Case study I: Compounds present in the phenylalanine metabolism KEGG pathway

Name	Unique identifier KEGG ID	Run Time (sec)
Pyruvate	KGID_C00022	107
Acetyl-CoA	KGID_C00024	756
Succinate	KGID_C00042	191
L-Phenylalanine	KGID_C00079	322
L-Tyrosine	KGID_C00082	390
Malonyl-CoA	KGID_C00083	656
Acetaldehyde	KGID_C00084	21
Succinyl-CoA	KGID_C00091	614
Fumarate	KGID_C00122	107
4-Hydroxybenzoate	KGID_C00156	79
Phenylpyruvate	KGID_C00166	200
Benzoate	KGID_C00180	71
p-Coumaroyl-CoA	KGID_C00223	994
Caffeoyl-CoA	KGID_C00323	999
Feruloyl-CoA	KGID_C00406	946
trans-Cinnamate	KGID_C00423	239
S-Benzoate	KGID_C00512	836
Phenylacetyl-CoA	KGID_C00582	835
2-Hydroxy-2,4-pentadienoate	KGID_C00596	161
Phenylacetaldehyde	KGID_C00601	122
2-Methylpropanoyl-CoA	KGID_C00630	736
4-Hydroxyphenylacetate	KGID_C00642	246
4-Hydroxy-3-methoxy-benzaldehyde	KGID_C00755	160
Salicylate	KGID_C00805	111
4-Coumarate	KGID_C00811	248
3-(2-Hydroxyphenyl)propanoate	KGID_C01198	384
Ephedrine	KGID_C01575	371
Hippurate	KGID_C01586	336
trans-2-Hydroxycinnamate	KGID_C01772	279
alpha-Oxo-benzeneacetic	KGID_C02137	112
3-Oxoadipyl-CoA	KGID_C02232	709
D-Phenylalanine	KGID_C02265	414
2-Phenylacetamide	KGID_C02505	131
2-Hydroxy-3-phenylpropenoate	KGID_C02763	256
(+)-Pseudoephedrine	KGID_C02765	369

N-Acetyl-L-phenylalanine	KGID_C03519	441
4-Hydroxy-2-oxopentanoate	KGID_C03589	399
3-(2,3-Dihydroxyphenyl)propanoate	KGID_C04044	410
Phenylacetylglutamine	KGID_C04148	433
(R)-2-Methylimino-1-phenylpropan-1-ol	KGID_C04351	327
2-Hydroxy-6-oxonona-2,4-diene-1,9-dioate	KGID_C04479	399
Phenethylamine	KGID_C05332	103
3-Hydroxyphenylacetate	KGID_C05593	250
Phenylacetylglycine	KGID_C05598	408
Phenyllactate	KGID_C05607	323
N-Acetyl-D-phenylalanine	KGID_C05620	434
Phenylpropanoate	KGID_C05629	226
2-Hydroxyphenylacetate	KGID_C05852	199
Phenylethyl	KGID_C05853	111
2,6-Dihydroxyphenylacetate	KGID_C06207	190
Capsaicin	KGID_C06866	122
Phenylacetic	KGID_C07086	141
4-Hydroxy-3-methoxyphenyl-beta-hydroxypropanoyl-CoA	KGID_C07303	1360
D-Cathine	KGID_C08300	308
D-Cathinone	KGID_C08301	178
3-(3-Hydroxyphenyl)propanoic	KGID_C11457	420
cis-3-(Carboxy-ethyl)-3,5-cyclo-hexadiene-1,2-diol	KGID_C11588	387
trans-3-Hydroxycinnamate	KGID_C12621	267
cis-3-(3-Carboxyethenyl)-3,5-cyclohexadiene-1,2-diol	KGID_C12622	411
trans-2,3-Dihydroxycinnamate	KGID_C12623	386
2-Hydroxy-6-ketononatrienedioate	KGID_C12624	488
5-Carboxy-2-pentenoyl-CoA	KGID_C14144	681
(3S)-3-Hydroxyadipyl-CoA	KGID_C14145	759
Phenylglyoxylyl-CoA	KGID_C15524	933
Vanillylamine	KGID_C16666	127
(-)-Norephedrine	KGID_C16719	257
Pyruvophenone	KGID_C17268	169
8-Methyl-6-nonenoic	KGID_C18202	351
3-Oxo-5,6-dehydrosuberyl-CoA	KGID_C19945	815
3-Oxo-5,6-dehydrosuberyl-CoA	KGID_C19946	874
2-Oxepin-2(3H)-ylideneacetyl-CoA	KGID_C19975	853
2-(1,2-Epoxy-1,2-dihydrophenyl)acetyl-CoA	KGID_C20062	723

Table S4: Case study II: Top 200 prescribed drugs in USA in 2011

Name	Unique Identifier ChemSpider	Unique Structures	Run Time* (sec)
Abilify	CSID_54790	1	544
Actos	CSID_54590	2	513
Advair Diskus	CSID_7987322	2	778
Albuterol	CSID_1999	1	439
Allopurinol	CSID_2010	1	52
Alprazolam	CSID_2034	1	96
Amitriptyline HCl	CSID_10594	2	460
Amlodipine Besylate	CSID_54537	2	579
Amoxicillin	CSID_31006	1	601
Amphetamine Salts	CSID_13852819	1	99
Atenolol	CSID_2162	1	416
Azithromycin	CSID_10482163	1	465
Bactrim	CSID_318412	2	154
Benicar	CSID_115748	1	738
Benicar HCT	CSID_139674	1	530
Boceprevir	CSID_8499830	1	501
Buprenorphine HCl	CSID_2297864	2	568
Bystolic	CSID_8108633	2	501
Carisoprodol	CSID_2478	1	250
Carvedilol	CSID_2487	1	111
Celebrex	CSID_2562	1	111
Celexa	CSID_70381	2	379
Cephalexin	CSID_25541	1	531
Cheratussin AC	CSID_56541	1	59
Cheratussin AC	CSID_58641	1	697
Cialis	CSID_99301	1	694
Ciprofloxacin HCl	CSID_56700	3	459
Clindamycin HCl	CSID_10482112	2	344
Codeine Sulfate	CSID_2341112	2	506
Crestor	CSID_4445607	3	234
Cyclobenzaprine Hydrochloride	CSID_21168	2	313
Cymbalta	CSID_54822	1	369
Diazepam	CSID_2908	1	282
Digoxin	CSID_2006532	1	397
Diovan	CSID_54833	1	459
Diovan HCT	CSID_7986336	2	543
Dyazide	CSID_56657	2	152

Efexor	CSID_56641	2	354
Enalapril Maleate	CSID_21112356	2	522
Endocet/Oxycontin	CSID_4447649	1	493
Famotidine	CSID_3208	1	326
Flovent HFA	CSID_392059	1	427
Fluconazole	CSID_3248	1	164
Fluoxetine HCl	CSID_56589	2	375
Folic Acid	CSID_5815	1	562
Furosemide	CSID_3322	1	502
Gabapentin	CSID_3328	1	308
Glipizide	CSID_3359	1	584
Glyburide	CSID_3368	1	828
Hydrochlorothiazide	CSID_3513	1	115
Ibuprofen (Rx)	CSID_3544	1	408
Januvia	CSID_4953630	2	460
Levaquin	CSID_131410	1	714
Levothyroxine Sodium	CSID_56705	2	63
Lexapro	CSID_10616991	2	416
Lidoderm	CSID_3548	1	415
Lisinopril	CSID_4514932	3	417
Loestrin 24 Fe	CSID_5770	1	414
Lorazepam	CSID_3821	1	422
Losartan Potassium	CSID_54768	2	22
Lovastatin	CSID_48085	1	358
Lovaza	CSID_8007146	2	712
Lyrica	CSID_4589156	1	340
Meloxicam	CSID_10442740	1	386
Metformin HCl	CSID_13583	2	95
Methylprednisolone	CSID_6485	1	400
Metoprolol Succinate	CSID_56654	3	928
Metoprolol Succinatee	CSID_4027	1	404
Metoprolol Tartrate	CSID_390070	3	1058
Naloxone HCl	CSID_4576530	2	468
Namenda	CSID_157849	2	113
Naproxen	CSID_137720	1	220
Nasonex	CSID_390091	1	440
Nexium	CSID_7843322	3	42
Niaspan	CSID_913	1	10
Nuvaring	CSID_8136308	2	775
Omeprazole (Rx)	CSID_4433	1	266
Oxycodone HCl	CSID_4575389	2	479

Pantoprazole Sodium	CSID_5293370	2	21
Paroxetine HCl	CSID_23089260	2	439
Penicillin VK	CSID_8286	2	58
Percocet	CSID_4881971	3	639
Plavix	CSID_54632	1	430
Pravastatin Sodium	CSID_49400	2	142
Prednisone	CSID_5656	1	415
Prednisone	CSID_4642486	1	379
Premarin	CSID_9532	2	52
Premarin	CSID_570974	2	54
Proair HFA	CSID_36448	3	850
Promethazine HCl	CSID_5792	2	128
Risperidone	CSID_4895	1	551
Seroquel	CSID_4444493	3	1017
Simvastatin	CSID_49179	1	363
Singulair	CSID_4444508	2	52
Spiriva Handihaler	CSID_10482095	2	356
Symbicort	CSID_36566	1	443
Tamsulosin HCl	CSID_4515016	2	143
Tramadol HCl	CSID_56711	2	398
Trazodone HCl	CSID_56652	2	387
Triamcinolone Acetonide	CSID_6196	1	429
Tricor	CSID_3222	1	460
Tri-Sprintec / TriNessa	PCID_9571023	2	788
Viagra	CSID_56586	2	955
Vicodin	CSID_4576477	3	766
Vicodin	CSID_4677998	3	900
Vicodin	CSID_4881954	10	1671
Vicodin	CSID_21230266	3	923
Vitamin D (Rx)	CSID_4444353	1	360
Vytorin	CSID_8008151	2	981
Vyvanse	CSID_9772457	3	321
Warfarin Sodium	CSID_10442445	1	564
Zantac	CSID_43590	2	375
Zestoretic	CSID_21106405	2	497
Zetia	CSID_132493	1	652
Zolpidem Tartrate	CSID_390093	3	1129
Zyprexa	CSID_10442212	1	318

* The run time is the total time to perform *in-silico* fragmentation on all the unique structures of the corresponding drug.

Spectral Matching Using the Modified pMatch Algorithm (Ye, et al., 2010)

The pMatch algorithm, utilizing a novel probability based model to score spectral comparison, is reported to obtain better identification performance than conventional methods. The original algorithm is designed for mass spectrometry based protein identification. In this work we propose a modified pMatch algorithm.

1. Preprocessing

A series of preprocessing filters are applied before spectral matching: Intensity value filter removes peaks that have a relative intensity smaller than a given threshold; Intensity number filter retains a given number of the most intensive peaks; Isotope filter removes isotopic peaks. Preprocessing helps the algorithm to improve matching accuracy and reduce computational time.

2. Peak Matching

The original pMatch algorithm takes two types of peak matching into account: S1 denotes accurate matching, and S2 denotes matching with mass shifts referring to the precursor ion mass difference. The precursor mass difference, caused by peptides with unusual post-translational modifications (PTMs), is not relevant for metabolomics. In the modified pMatch algorithm only the accurate peak matching is therefore considered.

Before matching, the precursor ion mass of the real spectrum is compared to that of each compound in the *in-silico* library. Compounds with precursor mass difference smaller than a given tolerance T_p are retained in a candidate set $\boldsymbol{C}$ for further matching and scoring.

$$\boldsymbol{C} = \{\textit{in-silico} \text{ compound } c_i \text{ with precursor m/z value } m_P\text{: } |m_P - m_R| < T_p\}$$

where c_i is the ith compound in the *in-silico* library, and m_R is the precursor ion mass of the real spectrum. Peaks in the real spectrum are sorted in the descending order of their intensities, and determine their hits in each candidate compound. The peak hits in candidate compound c_i are:

$$\boldsymbol{S}_i = \{\textit{in-silico} \text{ peaks in } c_i \text{ with m/z value } m_L\text{: } |m_L - m_Q| < T_p\},\ c_i \in \boldsymbol{C}$$

in which m_Q is the *m/z* value of the explained real peak. Each peak in a candidate *in-silico* compound can only be matched at most once.

3. Similarity Scoring

In the modified pMatch algorithm, three sub-scores are employed to measure the spectral similarity: (1) spectral dot-product score (SDP_Score), (2) probability-based score (P_Score), and (3) matching distance score (MD_Score). These sub-scores and the overall similarity score are calculated for each candidate *in-silico* compound.

(1) SDP_Score: the SDP_Score for candidate compound c_i is calculated using the following equation:

$$\text{SDP\_Score} = \frac{\sum_{peaks\_in\_\boldsymbol{S}_i} I_Q \times I_L}{\sqrt{\sum_{real\_peaks} I_Q^2} \times \sqrt{\sum_{in-silico\_peaks} I_L^2}}$$

where I_Q and I_L are the intensities of real and *in-silico* peaks respectively, and *peaks_in_*$\boldsymbol{S}_i$ denotes the *in-silico* peaks in hits set $\boldsymbol{S}_i$. Intensity values for all the *in-silico* peaks are set to 100 (maximum relative intensity value of the real spectrum).

(2) P_Score: in pMatch algorithm, peaks in real spectrum with intensity values no less than 5% of the most intensive peak are defined as the capital peaks, and the mighty hits are matches between the capital peaks and the explained *in-silico* peaks. The global average probability of a mighty hit is defined as:

$$p = \frac{\sum_{i=1}^{C_{max}} k_i / n}{\sum_{i=1}^{C_{max}} m_i}$$

where n is the number of capital peaks in real spectrum, variables k_i and m_i are the numbers of mighty hits and all the hits in candidate compound c_i respectively, and C_{max} is the number of candidate compounds. The probability of at least one hit in m_i is a mighty hit is:

$$P_i = 1 - (1 - p)^{m_i}$$

Thereby the P_Score of candidate compound c_i is calculated using the following equation:

$$\text{P\_Score} = \sqrt{-\log\left(\sum_{j=k_i}^{n} C_n^j \cdot P^j \cdot (1-P)^{n-j}\right)}$$

(3) MD_Score: in the original pMatch algorithm, only the information of intensities and peak hits numbers is considered in scoring. In this work we introduce the MD_Score, containing the information of matching distances between the real spectrum and *in-silico* spectrum (candidate compound), into the modified algorithm to improve identification performance. The weighted matching distance in candidate compound c_i is defined as:

$$w_i = \sum_{peaks\_in\_\boldsymbol{S}_i} I_Q \cdot |m_Q - m_L|$$

where $\boldsymbol{S}_i$ is the peak hits set of c_i, variable I_Q, m_Q and m_L are the real intensity, real *m/z* value, and explained *in-silico* m/z value of each peak in the $\boldsymbol{S}_i$. The MD_Score is calculated using the following equation:

$$\text{MD\_Score} = 1 - \frac{w_i - w_{min}}{w_{max} - w_{min}}$$

in which w_{min} and w_{max} are the minimum and maximum value of w_i in all the candidate compounds respectively.

(4) Overall Similarity Score: the final similarity score is defined as the product of SDP_Score, P_Score, and MD_Score:

Overall similarity score = SDP_Score × P_Score × MD_Score

The higher the overall score, the more similar the real spectrum is compared to the candidate i*n-silico* spectrum (compound).

Table S5: Fragmentation spectra retrieved from MassBank (Horai, et al., 2010)

Case Study	Name	Unique Identifier MassBank	Number of Peaks	Record Title
**Phenylalanine metabolism KEGG pathway**	Acetyl-CoA	KNA00207	53	LC-ESI-ITFT; MS2; m/z:405.57; POS
	Capsaicin	WA001605	15	LC-ESI-Q; MS; POS; 30 V
	Isobutyryl-CoA	PR100154	14	LC-ESI-QTOF; MS2; CE:30 V; [M+H]+
	N-Acetyl-L-phenylalanine	KO002200	31	LC-ESI-QQ; MS2; CE:30 V; [M+H]+
	Succinic acid	KZ000074	82	GC-EI-TOF; MS; 2 TMS; BP:147
**Top 200 prescribed drugs in USA in 2011**	Amoxicillin	WA001751	112	LC-ESI-Q; MS; POS; 30 V
	Digoxin	WA000563	71	LC-ESI-Q; MS; POS; 30 V
	Meloxicam	WA002576	12	LC-ESI-Q; MS; POS; 30 V
	Naproxen	WA000359	13	LC-ESI-Q; MS; POS; 30 V
	Prednisone	CO000368	182	LC-ESI-QTOF; MS2; CE:30 eV;

Table S6: Results of the spectral matching using a mass tolerance (Tp) of 1 Da.

Case Study/*In-silico* library	Name (Unique Identifier MassBank) *	Candidate *In-silico* Compounds **	Overall Similarity Score	Explained Peaks (Percentage)	Total Distance (Absolute)
**Phenylalanine metabolism KEGG pathway**	Acetyl-CoA (KNA00207)	KGID_C00024	0.06907	48 (90.6%)	7.007
	Capsaicin (WA001605)	KGID_C06866	0.13561	13 (86.7%)	2.129
	Isobutyryl-CoA (PR100154)	KGID_C00630	0.05078	12 (85.7%)	0.662
	N-Acetyl-L-phenylalanine (KO002200)	KGID_C03519	0.04375	29 (93.5%)	5.456
		KGID_C05620	0.04375	29 (93.5%)	5.456
	Succinic acid (KZ000074)	KGID_C00042	0.02951	31 (37.8%)	4.263
**Top 200 prescribed drugs in USA in 2011**	Amoxicillin (WA001751)	CSID_31006	0.21531	93 (83.0%)	3.336
	Digoxin (WA000563)	CSID_2006532	0.17972	42 (59.2%)	9.460
	Meloxicam (WA002576)	CSID_10442740	0.07393	6 (50.0%)	1.035
	Naproxen (WA000359)	CSID_137720	0.18800	5 (38.5%)	1.203
	Prednisone (CO000368)	CSID_5656	1.81681	175 (96.2%)	17.199
		CSID_4642486	1.81681	175 (96.2%)	17.199

* Fragmentation spectra retrieved from MassBank (Horai, et al., 2010).
** Includes all candidate *in-silico* compounds with an overall score larger than zero.

References

Horai, H., *et al.* (2010) MassBank: a public repository for sharing mass spectral data for life sciences, *Journal of Mass Spectrometry*, **45**, 703-714.

Ye, D., *et al.* (2010) Open MS/MS spectral library search to identify unanticipated post-translational modifications and increase spectral identification rate, *Bioinformatics*, **26**, i399-406.
